# The Role of Muscle Loading on Bone (Re)modeling at the Developing Enthesis

**DOI:** 10.1371/journal.pone.0097375

**Published:** 2014-05-21

**Authors:** Alexander M. Tatara, Justin H. Lipner, Rosalina Das, H. Mike Kim, Nikunj Patel, Eleni Ntouvali, Matthew J. Silva, Stavros Thomopoulos

**Affiliations:** Department of Orthopaedic Surgery, Washington University, St. Louis, Missouri, United States of America; Faculté de médecine de Nantes, France

## Abstract

Muscle forces are necessary for the development and maintenance of a mineralized skeleton. Removal of loads leads to malformed bones and impaired musculoskeletal function due to changes in bone (re)modeling. In the current study, the development of a mineralized junction at the interface between muscle and bone was examined under normal and impaired loading conditions. Unilateral mouse rotator cuff muscles were paralyzed using botulinum toxin A at birth. Control groups consisted of contralateral shoulders injected with saline and a separate group of normal mice. It was hypothesized that muscle unloading would suppress bone formation and enhance bone resorption at the enthesis, and that the unloading-induced bony defects could be rescued by suppressing osteoclast activity. In order to modulate osteoclast activity, mice were injected with the bisphosphonate alendronate. Bone formation was measured at the tendon enthesis using alizarin and calcein fluorescent labeling of bone surfaces followed by quantitative histomorphometry of histologic sections. Bone volume and architecture was measured using micro computed tomography. Osteoclast surface was determined via quantitative histomorphometry of tartrate resistant acid phosphatase stained histologic sections. Muscle unloading resulted in delayed initiation of endochondral ossification at the enthesis, but did not impair bone formation rate. Unloading led to severe defects in bone volume and trabecular bone architecture. These defects were partially rescued by suppression of osteoclast activity through alendronate treatment, and the effect of alendronate was dose dependent. Similarly, bone formation rate was increased with increasing alendronate dose across loading groups. The bony defects caused by unloading were therefore likely due to maintained high osteoclast activity, which normally decreases from neonatal through mature timepoints. These results have important implications for the treatment of muscle unloading conditions such as neonatal brachial plexus palsy, which results in shoulder paralysis at birth and subsequent defects in the rotator cuff enthesis and humeral head.

## Introduction

The development of a functional musculoskeletal system requires muscle forces [1], [2], [3], [4]. It is well established that loading regulates the shape and density of developing bones and the size and force-generating capacity of developing muscles. The absence of muscle loads during fetal development retards mineralization and leads to malformed bone [3]. Similarly, the growth and development of mineralized tissues is significantly impaired if muscle load is removed post-natally [5], [6], [7]. However, little is known about the force-mediated regulation of the developing tendon-to-bone attachment (the “enthesis”), despite its critical role in the transfer of muscle forces to bone for subsequent joint motion [8], [9].

We previously showed in a mouse animal model that shoulder paralysis at birth leads to bony and soft tissue defects by 28 days post-natally, including impaired bony architecture and decreased enthesis mechanical properties [5], [6], [7], [10], [11]. The bone- and joint-level deficiencies closely mimicked the clinical condition neonatal brachial plexus palsy, which results from injury to the brachial plexus during difficult childbirth and affects up to 1 in 250 infants [12], [13], [14]. During post-natal development of the shoulder in normal loading conditions, high osteoclast numbers were seen at the tendon enthesis, presumably due to the high rate of bone formation [5]. Osteoclast numbers decreased steadily with increasing post-natal age. However, in the absence of muscle load, osteoclast numbers remained elevated, possibly maintaining high levels of resorption and preventing mineral accumulation in the developing enthesis and adjacent bone. These defects in bone (re)modeling led to dramatic impairment of enthesis biomechanics [10], [11].

Skeletal disuse in mature animals leads to both diminished bone formation and elevated bone resorption [15]. Our previous results in the developing enthesis implied that defects from paralysis were partly caused by increased resorption; however, bone formation was not measured. Our observations therefore led to the question: were the unloading-induced developmental defects (e.g., bony architecture, mineralization, enthesis biomechanics) due to suppressed bone formation, elevated bone resorption, or both? More generally, were these defects due to defective bone modeling (shaping of bone where resorption and formation are uncoupled), remodeling (coupled action of bone resorption and formation on the same bone surface), or both? To explore these questions, muscle load was removed in the current study using muscle paralysis at birth; osteoclast activity was modulated using bisphosphonate treatment; and bone formation was measured using fluorescent labeling of bone surfaces. We hypothesized that unloading via muscle paralysis would suppress bone formation and enhance bone resorption at the enthesis, and that the bony defects could be rescued by suppressing osteoclast activity. Our results indicated that muscle unloading does not impair bone formation rate. Rather, it delays the initiation of endochondral ossification. Suppression of osteoclast activity partially rescued the bony defects caused by muscle unloading, implicating osteoclast activity as one cause of impaired enthesis development.

## Materials and Methods

### Ethics Statement

The animal procedures and care used in this study were approved by the institutional Animal Studies Committee at Washington University (Approval Number: 20130034A3) and carried out in strict accordance with the recommendations in the Guide for the Care and Use of Laboratory Animals of the National Institutes of Health.

### Animal Model

**Figure 1:**
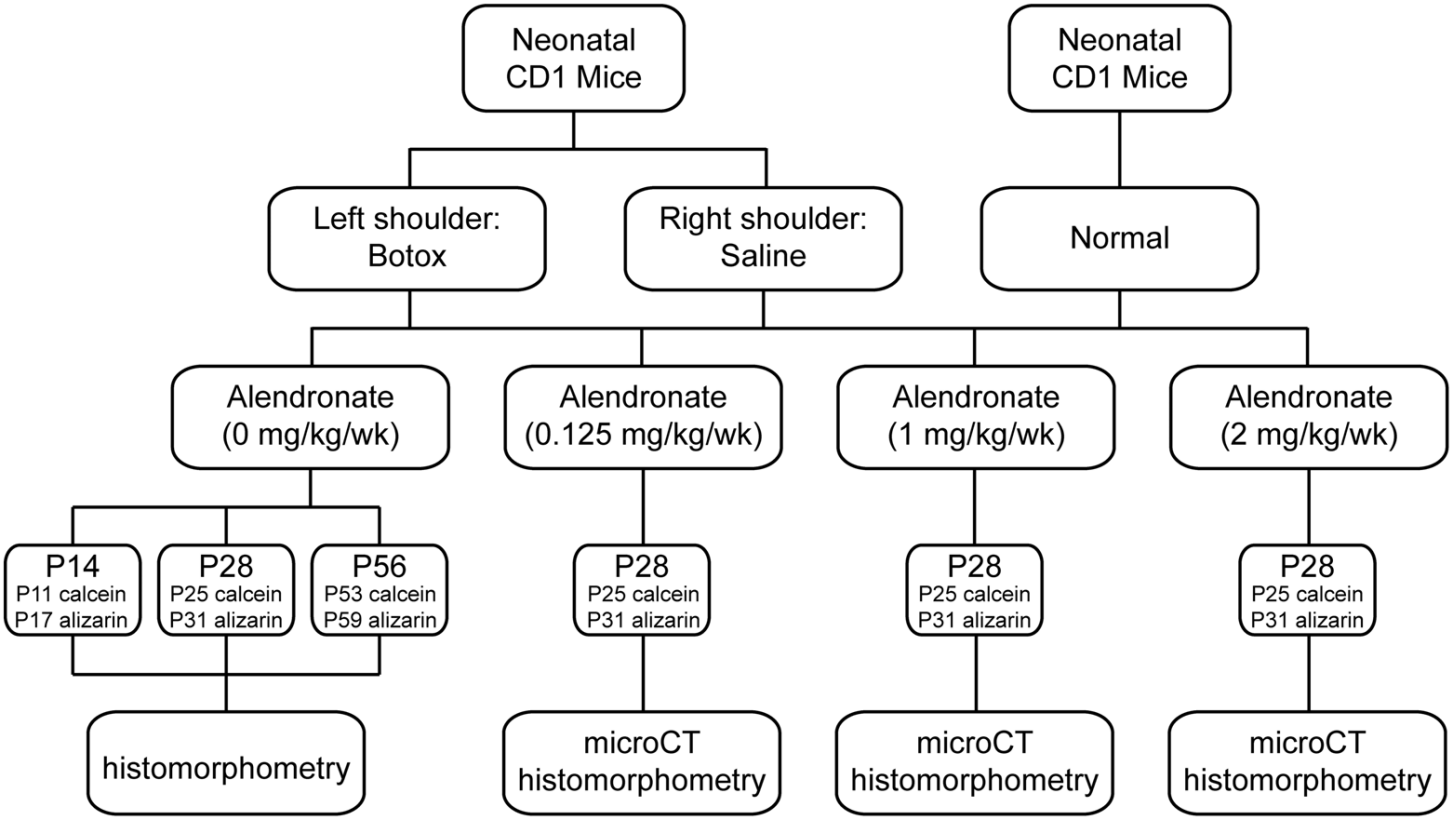
The study design consisted of Botox, Saline, and Normal groups treated with varying doses of alendronate and examined at three post-natal (P) timepoints.

In order to remove muscle loading across the shoulder during post-natal development, the left supraspinatus muscles (“Botox” group) of neonatal CD-1 mice were injected with 10 µL of 0.2 U of botulinum toxin A (BOTOX, Allergen, Inc.) (Figure 1) [5], [6], [7]. The right supraspinatus muscles received saline injections (“Saline” group) and served as contralateral controls. Paralysis was maintained from birth through sacrifice. The injections were started at day 1 after birth and repeated twice a week to maintain paralysis through P28. From P28 through 56, injections were repeated once a week to maintain paralysis. Previous studies established an effective dose of 0.05 U per gram body weight, delivered in 10 µL volume with a 30 gauge needle intramuscularly into the supraspinatus muscle. A separate group of animals was allowed to develop without saline or botulinum toxin injections (“Normal” group).

### Bone Formation

**Figure 2:**
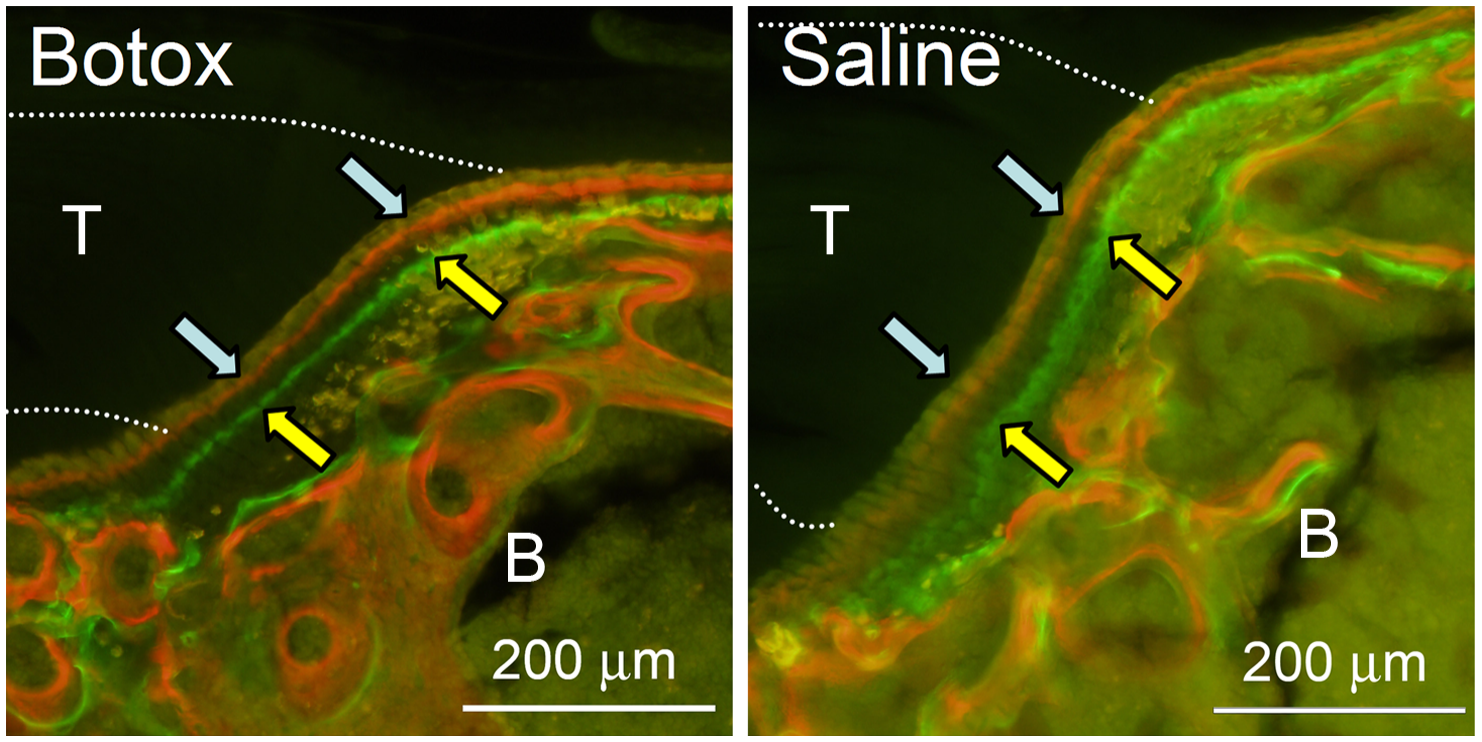
Alizarin (blue arrows) and calcein (yellow arrows) labels at the supraspinatus enthesis for representative Botox and Saline sections at P28. The dotted lines extending to the left in each panel indicate the approximate location of the supraspinatus tendon (T). Trabecular bone and the epiphysis of the humeral head can be seen to the right of the entheses (B).

In order to examine the effect of unloading on bone formation at the enthesis during development, mice were separated into three time points (post-natal days P14, P28, and P56). Botox/Saline mice (n = 8–10) and Normal mice (n = 8–11) were examined at each timepoint. Bone formation was determined based on injections of labels before and after these timepoints of interest. In this manner, the measurement of changes between the two fluorescent labels described cumulative bone formation centered around the timepoint of interest. Mice were given intraperitoneal injections of calcein green (5 mg/kg, Sigma-Aldrich) and alizarin-complexone (30 mg/kg, Sigma) 3 days before and 3 days after the given time point, respectively (Figure 2). 7 days after the given time point, the animals were sacrificed. Incorporation of the two labels could then be used to study bone formation a timepoints centered around P14 (i.e., P11–P17), P28, (i.e., P25–P31), and P56 (i.e., P53–P59).

### Inhibition of Osteoclast Activity

To clarify the role of osteoclasts in unloading-induced bone resorption, the bisphosphonate alendronate (Sigma-Aldrich) was administered weekly through intraperitoneal injections to mice [16]. Four doses (no dose = 0 mg/kg/week, low dose = 0.125 mg/kg/week, medium dose = 1 mg/kg/week, and high dose = 2 mg/kg/week alendronate in PBS) were examined in Normal and Botox mice (N = 10 for the no dose/Normal group, N = 13–17 per dose for all other groups). At P28, 5–7 mice from each group were sacrificed and analyzed for osteoclast surface by quantitative histomorphometry, and bone volume, bone architecture, and muscle volume by micro computed tomography (µCT), as described below. All other mice were given intraperitoneal injections of calcein green at P25 and intraperitoneal injections of alizarin red at P31 (i.e., 3 days before and 3 days after P28). At P35, the mice were sacrificed.

### Sample Preparation and Measurement

Humerus-enthesis-muscle samples were dissected, fixed in 10% formalin, and dehydrated to 70% ethanol. P28 samples allocated to bone morphometry and TRAP staining were scanned in air by µCT (µCT 40; Scanco Medical, Basserdorf, Switzerland; 55 kV, 145 µA, 20 µm voxel, 99 msec integration, 250 projections) at room temperature as described previously [17]. Briefly, supraspinatus muscle volume was measured from the most medial end of the scapular origin to the insertion on the humeral head. The volume of bone from the distal end to the anatomical neck of the superior end of the humeral head was measured for mineralized bone. This resulted in a region of interest encompassing the mineralized tissue at the enthesis and the trabecular bone directly adjacent to the enthesis up to the growth plate. The trabecular thickness, number, and separation were measured to evaluate the bone architecture. To determine trabecular parameters, the endosteal margin of the humeral head was defined using hand drawn contours followed by thresholding to separate bone from background (sigma = 0, support = 0, lower/upper threshold = 270/1000 = 332 mg HA/cm^3^, peel iteration = 4). Standard outcomes included trabecular bone volume per tissue volume (BV/TV), trabecular number (Tb.N), thickness (Tb.Th), separation (Tb.Sp) and connectivity density (Conn.D).

After µCT scanning, specimens were paraffin embedded, sectioned at 5 µm, and stained for tartrate resistant acid phosphatase (TRAP). Specifically, slides were first incubated in a solution of sodium acetate, L-(+) tartaric acid, glacial acetic acid, naphthol AS-BI phosphate, ethylene glycol monoethyl ether, and distilled water for 45 min at 37°C. Slides were then incubated in a solution of sodium acetate, L-(+) tartaric acid, glacial acetic acid, sodium nitrite, pararosaniline chloride, 2N hydrochloric acid, and distilled water for 5 min at 37°C. Slides were then rinsed in water, counterstained with haematoxylin, dehydrated through graded alcohols, cleared with xylenes, and coverslipped. One randomly chosen section per specimen was analyzed by a single user (NP) with bright-field microscopy and Bioquant Osteo II software (Bioquant) for analysis of osteoclast surface on the bone surfaces adjacent to the enthesis at 16x objective magnification.

Samples allocated to bone formation histomorphometry were fully dehydrated and embedded in polymethylmethacrylate (Sigma). Two to three sections of thickness 100 µm were cut from each sample using a saw microtome (Leica Microsystems, SP 1600) and adhered onto glass slides. The sections were imaged using an inverted microscope with a 100 W mercury-halogen light under 10x objective (DP-30, Olympus). The calcein green marker was captured under a fluorescein isothiocyanate (FITC) filter and the alizarin red marker was captured using a tetramethylrhodamine isothiocyanate (TRITC) filter. The images were merged with Olympus software. A bright field image was taken for visualization of total bone surface. The calcein and alizarin-labeled bone surfaces were analyzed by a single user (AT) with Bioquant Osteo II software. Analyses were performed for the enthesis and for the trabecular bone adjacent to the enthesis (Figure 2). The values measured were averaged between sections to obtain single-labeled bone surface (sLS/BS), double-labeled bone surface (dLS/BS), mineralizing surface per bone surface (MS/BS), mineral apposition rate (MAR) and bone formation rate (BFR/BS) for each sample. MAR and BFR were only computed when there were double-labels [18]. A result of “n.d.” (“no data”) is indicated when an outcome was not computable.

### Statistics

Botox and Saline groups were compared using paired t-tests. Botox and Saline were compared to Normal and across time using an analyses of variance followed by a Fisher’s least squares difference post-hoc test. Significance was set at p<0.05.

## Results

### The Effect of Muscle Unloading on Bone Formation at the Developing Enthesis and Adjacent Trabecular Bone

**Figure 3:**
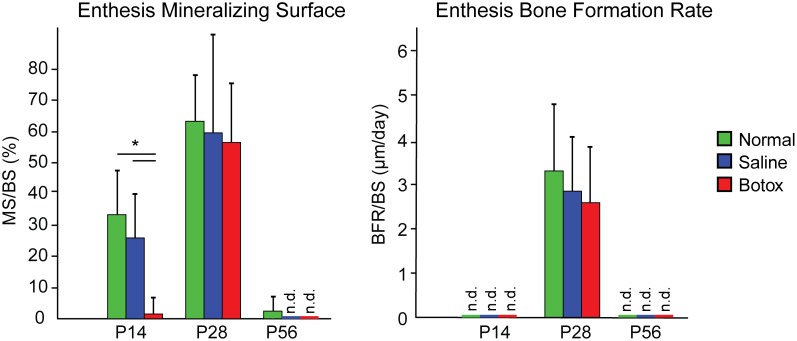
Bone formation histomorphometry at the developing enthesis. MS/BS was significantly lower in the Botox group compared to the Saline and Normal groups at P14 (*p<0.05). There was a significant increase in MS/BS from P14 to P28 followed by a significant decrease from P28 to P56.

Bone formation rate and mineralizing surface/bone surface at the enthesis was highest at P28 (Figure 3; Tables S1–S2 in file S1). During post-natal shoulder development, different fluorochrome labeling patterns were seen across time points and loading groups at the tendon enthesis. At P14, each specimen in the Saline and Normal groups showed only the second (red alizarin) label. This implies that mineralization began after the calcein was delivered (P11) but before the alizarin was delivered (P17). By contrast, only 1 specimen out of 18 in the Botox group showed any label at all at P14, signifying that mineralization was delayed at least past P17 in this group. At P28, all groups showed bands of calcein and alizarin. Importantly, muscle load did not affect bone formation outcomes at this timepoint. The mineral apposition rate was ∼2x higher and the bone formation rate was ∼5x higher in the enthesis compared to the trabecular bone adjacent to the enthesis (Figure 3; Tables S1–S2 in file S1). At P56, bands were rarely seen in any group (calcein bands were seen in 2 out of 8 samples in the normal group, 0 out of 9 samples in the saline group, and 0 out of 9 samples in the botox group). The lack of fluorophore update was likely due to the timing of the injections relative to the slow bone formation rates of this relatively mature timepoint.

### The Effect of Alendronate on the Unloaded Developing Enthesis and Adjacent Trabecular Bone

**Figure 4:**
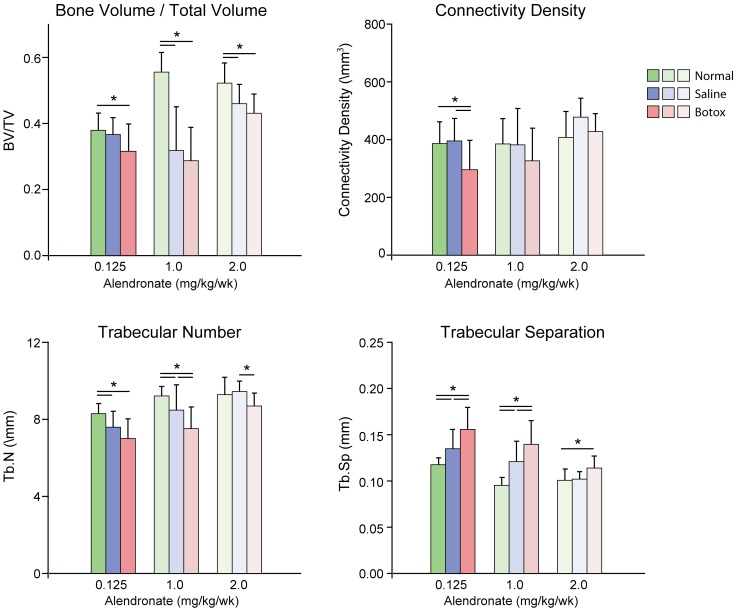
The effect of alendronate on bone architecture at P28 (*p<0.05). Alendronate dose and group (Botox vs. Saline vs. Normal) significantly affected BV/TV, connectivity density, Tb.N, and Tb.Sp. At the highest dose of alendronate, Botox was significantly different than Normal for BV/TV and Tb.Sp. Note that results for animals without alendronate treatment have been published previously [5].

**Figure 5:**
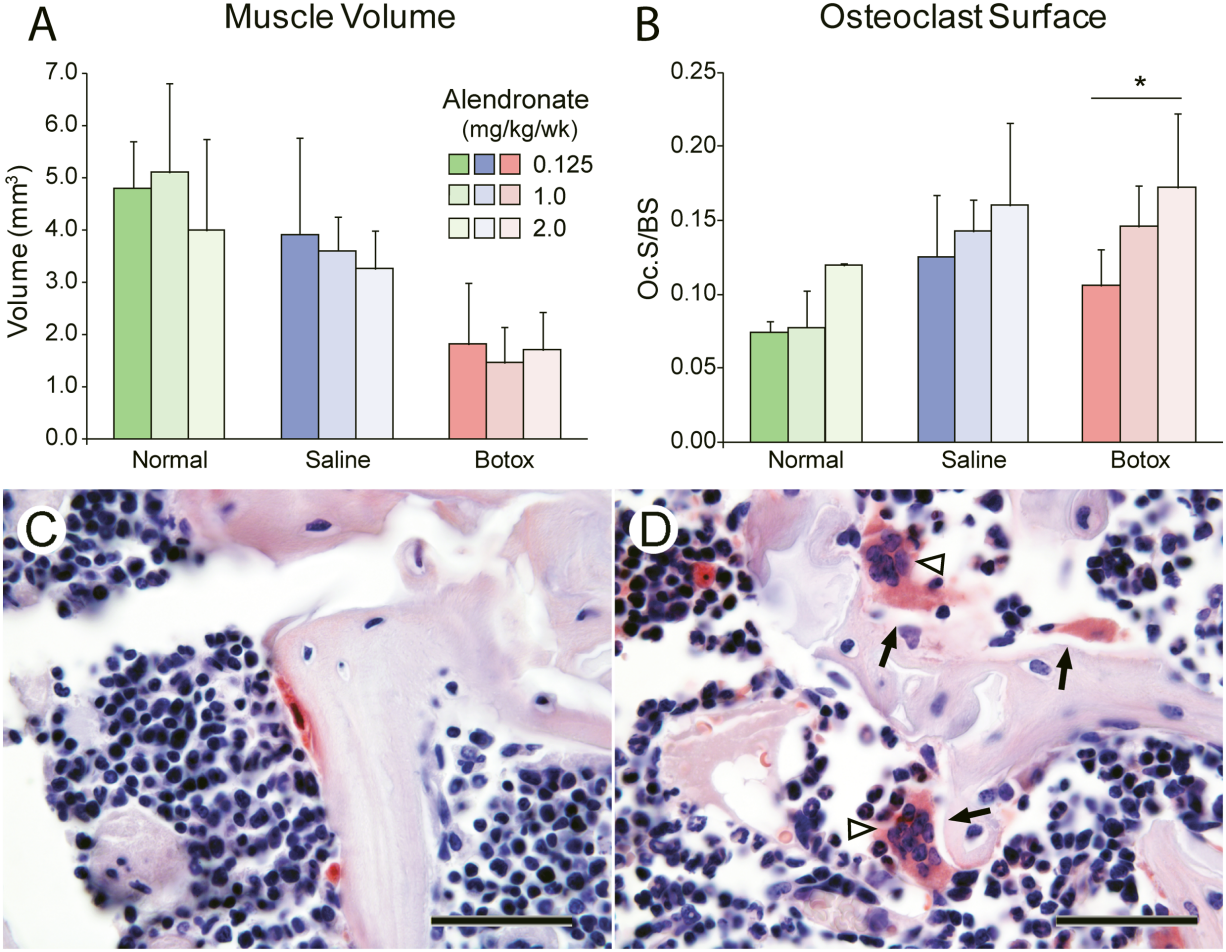
The effects of alendronate on muscle volume and osteoclasts. (**A**) There was no significant effect of alendronate on muscle volume at P28. Muscle volume was significantly reduced in the Botox group compared to the Saline and Normal groups. (**B**) Alendronate dose and group (Botox vs. Saline vs. Normal) significantly affected osteoclast surface at P28. The effect of alendronate was most pronounced in the Botox group (*p<0.05). (**C–D**) High magnification views of osteoclasts (stained in red) in the (C) Normal group with a low dose any of alendronate and (D) Botox group with a high dose of alendronate. Osteoclasts in the bones treated with high doses of alendronate were hypernucleated (white arrowheads) and were detached from the underlying bone (black arrows), suggesting abnormal function (100x objective, TRAP stain, scale bar = 20 µm).

Because the histomorphometry results across time (Figure 3) revealed that the tendon enthesis only absorbed both fluorochrome labels at P28, this time point was chosen to study the effects of alendronate. When examining the bone at the supraspinatus enthesis and the adjacent trabeculae, alendronate increased bone volume and trabecular number in a dose-dependent manner and reduced differences between the Normal and Botox groups (Figure 4; Figure S1 and Table S3 in file S1). It also increased connective density at medium and high doses. However, none of the alendronate dosages were able to completely return the unloaded tissues back to normal (Figure 4; Figure S1 and Table S3 in file S1). As expected, muscle volume was decreased due to paralysis and unaffected by alendronate (Figure 5). Alendronate increased osteoclast surface in a statistically significant, dose-dependent manner (Figure 5; Figure S2 in file S1). Many of the osteoclasts in the bones treated with medium and high doses of alendronate appeared abnormal (Figure 5). These cells were hypernucleated, lacked a lysosome zone, had pyknotic nuclei, did not show nuclear polarization away from the bone, and were detached from the underlying bone, suggesting abnormal function.

**Figure 6:**
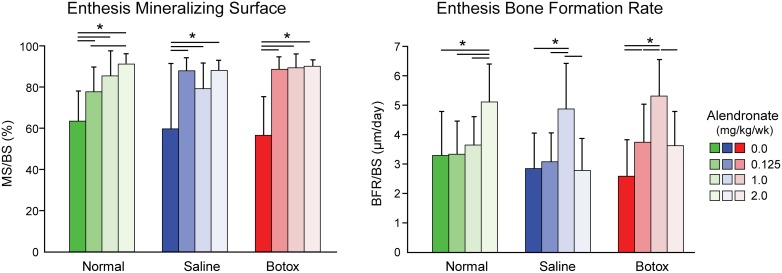
The effect of alendronate on bone formation histomorphometry at P28. There was a significant effect of alendronate on BFR/BS and MS/BS (*p<0.05).

At the tendon enthesis in normal animals, alendronate at its highest dose increased MAR, MS/BS, and BFR/BS at the enthesis and in the trabecular bone directly adjacent to the enthesis (Figure 6; Tables S4–S5 in file S1). However, in both the saline and botox specimens, alendronate had a biphasic effect in which it increased mineral apposition rate and bone formation rate at the medium dose but not at the high dose. Alendronate increased the mineralizing surface to total bone surface ratio equally regardless of dose. There was no significant effect of alendronate on mineral apposition rate of the trabecular bone at P28. In general, there was a trend for increasing dose to increase bone formation rate and mineralizing surface percentage. There were no statistically significant results across loading groups. Bone histomorphometry measures at the enthesis and adjacent bone were similar when comparing normal, saline, and botox for a particular alendronate dose (Figure 6; Tables S4–S5 in file S1).

## Discussion

The current study demonstrated that muscle loading plays an important role in the formation of a mineralized enthesis. Although muscle unloading did not significantly affect measurements such as bone formation rate and mineral apposition rate, muscle unloading did delay the initiation of the mineralizing front. This result implies that loading influences only the initial deposition of mineral at the enthesis, a process which is driven by endochondral ossification [19]. Similar bone formation rates were seen for loaded and unloaded tissues at later timepoints, suggesting that the differences in bone volume and trabecular architecture between the two groups were due to delayed accumulation of mineral and increased osteoclast activity, rather than decreased osteoblast activity. In support of this conclusion, treatment with the bisphosphonate alendronate partially rescued the bone phenotype resulting from muscle unloading.

We previously described the mineralization process at the developing rotator cuff tendon enthesis [19]. The transitional tissue between tendon and bone was examined from P7 through P56 using a variety of microscopic and spectroscopic techniques. Mineralizing cartilage originating in the secondary center of ossification of the humeral head epiphysis was evident at P7 and reached the tendon interface by P14. A mineral gradient was observed that spanned the length of one or two hypertrophic chondrocytes at the developing enthesis. P14 was also a critical timepoint in the current study; at this timepoint, a clear decrease in enthesis mineralizing surface was evident in the unloaded groups compared to the loaded groups. As the cells that form this mineralization front during this time frame are hypertrophic chondrocytes, not osteoblasts, we conclude that unloading affected chondrocyte hypertrophy and not osteoblast-mediated bone formation. In a previous study, we observed that hypertrophic chondrocytes remained at the enthesis through P21 in unloaded specimens whereas loaded controls no longer had hypertrophic chondrocytes after P14 [5]. Others have also demonstrated that mechanical load influences endochondral ossification [1], [2], [3], [4], [20]. It is possible that removing load delays hypertrophy and/or the migration of the mineralization front from the center of the humeral head to the tendon attachment. This is further supported by the lack of differences in bone formation rate (a measure primarily of osteoblast activity) at a later timepoint between loaded and unloaded tissues.

Bone formation of the trabeculae adjacent to the enthesis was similar to that of the enthesis. Although bone formation rates in the humeral head were unaffected by removal of load during post-natal growth, dramatic differences were noted in mineralizing surface and in accumulation of mineral. As with mineralization at the enthesis, these effects were likely due to a delay in the initiation of endochondral ossification (i.e., chondrocyte hypertrophy) and in high osteoclast activity. Previously, we showed that removal of load in the early post-natal period results in persistence of high osteoclast lined bony surfaces, whereas loaded tissues show a significant decline in osteoclast numbers over time [5]. In support of this, the current study showed that suppression of osteoclast activity via bisphosphonate treatment can partially rescue the bony defects caused by muscle unloading. Alendronate treatment led to increases in bone volume and connective density and in a recovery of trabecular architecture towards normal. These effects were dose-dependent, although complete restoration of normal properties was not achieved, even at the highest dose.

Treatment with alendronate led to increases in osteoclast surface in all groups in a dose dependent manner (Figure 5). This outcome appears contradictory to the observed increases in bone volume and the improvements in trabecular architecture in alendronate treated animals. Previously, it was believed that alendronate promoted apoptosis in osteoclasts and hence decreased their numbers [21], [22]. Decreased osteoclast numbers subsequently led to decreased resorption and increased bone mineral density. Recent reports, however, have demonstrated that apoptosis is not required for alendronate to effectively inhibit resorption [23]. Furthermore, using bone biopsies from a cohort of healthy post-menopausal women, it was demonstrated that treatment with alendronate for long periods of time is associated with *increases* in the number of osteoclasts [24]. Normal osteoclasts demonstrate tight cell-bone interfaces, lysosome-rich cytoplasm, and contain multiple nuclei polarized away from the bone. Osteoclasts in alendronate treated bones included giant hypernucleated cells lacking a lysosome zone and detached from the underlying bone, with pyknotic nuclei that were not polarized away from the bone. The authors suggested that these osteoclasts resorb bone poorly, if at all. The osteoclasts associated with alendronate treatment in the current study demonstrated similar morphologies.

Alendronate did not have an effect on bone formation properties in the trabecular bone across doses or loading regimes. However, at the enthesis in normal animals, alendronate at its highest dose increased mineral apposition rate, bone formation rate, and the mineralizing surface to total bone surface ratio. In both the Saline and Botox specimens, alendronate had a biphasic effect in which it increased mineral apposition rate and bone formation rate at the medium dose but not at the high dose. In contrast, it was previously reported that bisphosphonates can suppress osteoblast activity directly and through inhibition of their crosstalk with osteoclasts, including in unloading conditions [25], [26]. Alendronate may have led to increased available surface for mineralization by allowing osteoblasts access to surfaces that would otherwise have been occupied by Howship’s lacunae. Alternatively, bisphosphonates can also affect bone by directly preventing osteoblast and osteocyte apoptosis [27], [28], [29]. This effect is independent from its effect on osteoclasts and is mediated through connexin 43. It is unclear if alendronate directly influenced osteoblasts and osteocytes in the current study. Finally, it is also possible that there was a direct effect of alendronate on hypertrophic chondrocytes driving endochondral ossification at the tendon enthesis.

In the current study, both modeling and remodeling were considered due to the uncertainty associated with the mineralization process at the developing enthesis. Bone modeling shapes the bone during growth and, in some cases, during adaptation to changes in mechanical loading [30]. Resorption and formation are uncoupled during bone modeling. Bone remodeling, on the other hand, requires the coupled actions of bone resorption and formation on the same bone surface [30], [31]. In our previous study, we showed that the unloading-induced bone defects were likely osteoclast mediated, implying a remodeling process [5]. However, the results from the current study imply that mineralization at the enthesis is a modeling event, with few (if any) osteoclasts observed (Figure S2 in file S1). This is in contrast to the bone surfaces adjacent to the enthesis, where osteoclast activity was high. The effects and mechanisms of action of unloading and alendronate may therefore differ substantially between the entheses and the adjacent trabecular bone.

There were several limitations to the current study. First, although osteoclasts clearly played a dominant role in the unloading-induced defects at the developing enthesis, the mechanisms of bony defect formation remain unclear. Outcomes in the current study included quantification of bone formation and bony architecture, but did not include functional assays such as biomechanics. In addition, osteoclast activity was not directly measured (e.g., using serum markers) and osteoblast numbers were not determined. Also, while osteoclast activity was modulated using alendronate, osteoblast activity was not manipulated (e.g., using parathyroid hormone). A second limitation relates to our interpretation of missing label in young and unloaded animals. The lack of label could be due to delayed bone formation or remodeling of the incorporated label into new (unlabeled) bone. However, as the enthesis consists of unmineralized endochondral tissue at timepoints prior to P14, it is unlikely that calcein was incorporated and then rapidly remodeled in that time frame. Similarly, based on lower bone mineral in the unloaded groups, it is similarly unlikely that a calcein label was incorporated and then rapidly remodeled in those groups. A third limitation is the use of the saline injected contralateral shoulder as a control. As seen in our previous studies, there was often a significant change in this shoulder compared to normal shoulders [5], [6], [11]. This is attributed to behavioral changes in mice that have one paralyzed shoulder and not due to systemic toxic effects of the botulinum toxin, as neonatal neurotomy leads to similar developmental defects as chemical denervation [7]. Importantly, in the current study paralyzed shoulders were also compared to normal shoulders from a separate set of mice. A fourth limitation is the use of historical controls to draw conclusions for some outcomes. We previously reported µCT outcomes and osteoclast histomorphometry results for Botox, Saline, and Normal groups at P14, P28, and P56 (without alendronate treatment) [5]. Although direct statistical comparisons cannot be made use the previously published data, collected using slightly different techniques, the trends between groups in the previous study are consistent with the current study, especially at the lowest alendronate dose. Furthermore, the bone formation assays in the current study included a group that did not receive alendronate.

In summary, this study demonstrated that bone (re)modeling at the developing enthesis requires muscle forces. Using a mouse animal model of shoulder paralysis that mimics neonatal brachial plexus palsy, we determined that the developmental bony defects that result from muscle unloading are due primarily to elevated bone resorption, and not suppressed bone formation. Specifically, when suppressing osteoclast activity at the highest dose of alendronate, treated shoulders achieved 82% of normal BV/TV, 104% of normal connectivity density, 94% of normal trabecular number, and 88% of normal trabecular spacing (note that comparisons are made between groups for a particular dose to separate the effects of alendronate on normal growth from the effects of alendronate on botox-induced defects). Initiation of endochondral ossification in the developing humeral head was delayed in the absence of loading and suppression of osteoclast activity partially rescued the bony defects caused by muscle unloading. These results have important implications for the treatment of conditions such as neonatal brachial plexus palsy, a condition that results in shoulder paralysis at birth and subsequent bony defects in the humeral head.

## Supporting Information

File S1 **Figures S1–S2 and Tables S1–S5 can be found in the file S1.**
(DOCX)
