# Supplementary material for: The Role of Muscle Loading on Bone (Re)modeling at the Developing Enthesis
Source: PLoS One. 2014 May 21;9(5):e97375. doi: 10.1371/journal.pone.0097375 (PMC4029607; doi:10.1371/journal.pone.0097375)
Supplement: File S1 — Figures S1–S2 and Tables S1–S5 can be found in the file S1. (DOCX) [file pone.0097375.s001.docx]

**The role of muscle loading on bone (re)modeling**

**at the developing enthesis**

Alexander M. Tatara, B.S.

Justin H. Lipner, B.S.

Rosalina Das, M.S.

H. Mike Kim, M.D.

Nikunj Patel, B.S.

Eleni Ntouvali, M.D.

Mathew J. Silva, Ph.D.

Stavros Thomopoulos, Ph.D.

Department of Orthopaedic Surgery, Washington University, St. Louis, MO 63110

***- Supporting Information -***

Corresponding Author:

Stavros Thomopoulos, Ph.D.

Washington University

Department of Orthopaedic Surgery

660 South Euclid, Campus Box 8233

St. Louis, MO 63110

Phone: 314-362-8605

Fax: 314-362-0334

Email: ThomopoulosS@wudosis.wustl.edu

**Figure S1:** Representative μCT images for Normal, Saline, and Botox groups treated with alendronate at P28 (scale bar = 0.5 mm).


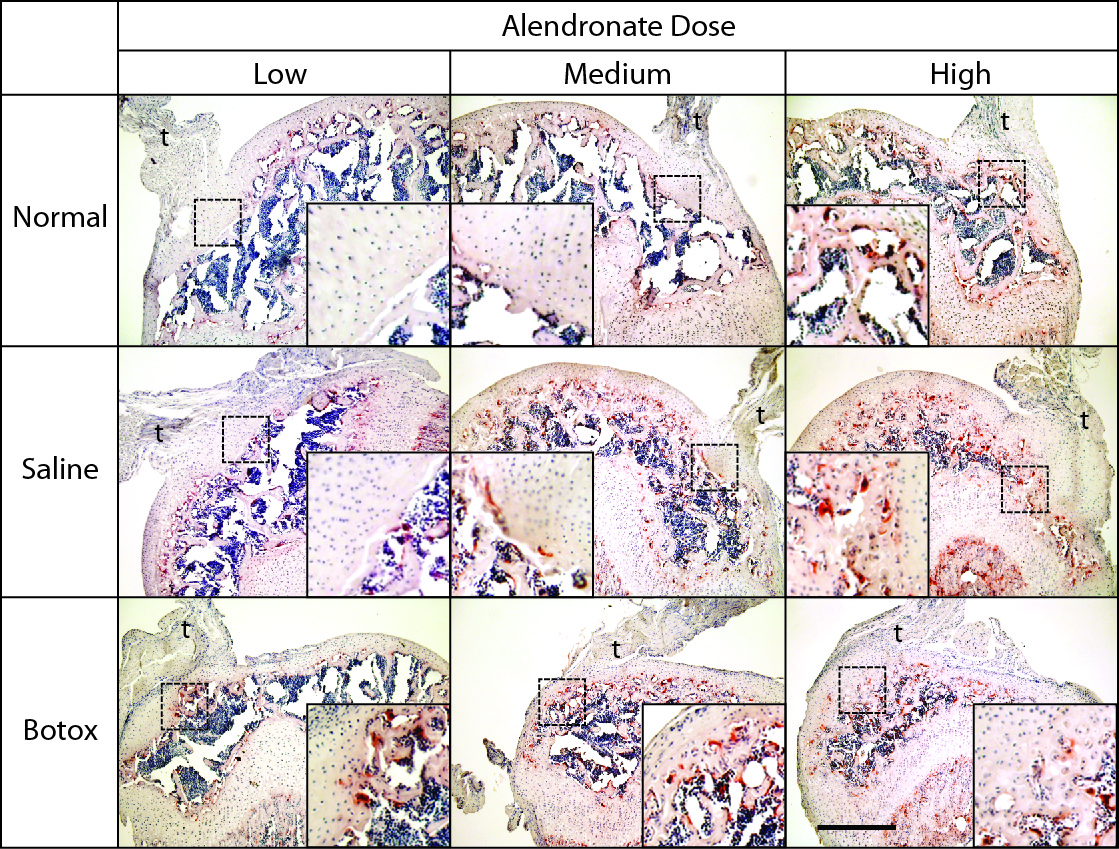


**Figure S2:** Representative TRAP stained histologic sections for Normal, Saline, and Botox groups treated with alendronate at P28 (4x objective, scale bar = 1 mm; insets show higher magnification versions of tendon entheses indicated by the dashed line boxes; all images were white balanced; t: tendon)

**Table S1:** Dynamic histomorphometry of tendon enthesis at P14, P28, and P56. Based on an ANOVA, there was a significant effect of time for SL/BS and DL/BS, and a significant effect of group for SL/BS. * p < 0.05 compared to normal at a particular timepoint.

| **Group** | **Timepoint** | **MAR (μm/day)** | **sL/BS (%)** | **dL/BS (%)** |
| --- | --- | --- | --- | --- |
| **Normal** | P14 | n.d. | 67.1 ± 27.0 | n.d. |
|  | P28 | 4.9 ± 1.7 | 20.7 ± 12.3 | 52.9 ± 16.8 |
|  | P56 | n.d. | 3.5 ± 6.8 | n.d. |
| **Saline** | P14 | n.d. | 51.1 ± 26.6* | n.d. |
|  | P28 | 4.1 ± 0.9 | 33.6 ± 20.4 | 53.5 ± 32.9 |
|  | P56 | n.d. | n.d. | n.d. |
| **Botox** | P14 | n.d. | 2.6 ± 7.2* | n.d. |
|  | P28 | 4.3 ± 1.6 | 21.8 ± 16.5 | 46.9 ± 15.9 |
|  | P56 | n.d. | n.d. | n.d. |

MAR Mineral Apposition Rate

BS Bone Surface

sL Single Labeled Surface

dL Double Labeled Surface

**Table S2:** Dynamic histomorphometry of trabecular bone directly adjacent to tendon enthesis at P14, P28, and P56. Based on an ANOVA, there was a significant effect of time for MAR, MS/BS, BFR/BS, SL/BS, DL/BS and a significant effect of group for MAR. * p < 0.05 compared to normal at a particular timepoint.

| **Group** | **Timepoint** | **MAR (μm/day)** | **MS/BS (%)** | **BFR/BS** | **sL/BS (%)** | **dL/BS (%)** |
| --- | --- | --- | --- | --- | --- | --- |
| **Normal** | P14 | 2.0 ± 0.3 | 19.9 ± 5.5 | 0.4 ± 0.1 | 22.4 ± 11.0 | 8.4 ± 4.3 |
|  | P28 | 2.2 ± 0.4 | 25.4 ± 7.5 | 0.6 ± 0.2 | 17.8 ± 6.1 | 16.5 ± 5.5 |
|  | P56 | 1.7 ± 0.3 | 17.8 ± 3.5 | 0.3 ± 0.1 | 20.3 ± 5.9 | 6.9 ± 2.8 |
| **Saline** | P14 | 1.6 ± 0.2* | 19.1 ± 7.0 | 0.3 ± 0.1 | 23.1 ± 13.0 | 7.4 ± 2.5 |
|  | P28 | 2.0 ± 0.3 | 26.2 ± 4.6 | 0.5 ± 0.1 | 16.0 ± 7.2 | 17.6 ± 6.9 |
|  | P56 | 1.4 ± 0.2 | 17.2 ± 3.2 | 0.2 ± 0.0 | 22.6 ± 6.6 | 5.7 ± 2.3 |
| **Botox** | P14 | 1.8 ± 0.3 | 17.1 ± 4.0 | 0.3 ± 0.1 | 23.4 ± 6.3 | 5.9 ± 1.7 |
|  | P28 | 2.0 ± 0.4 | 27.1 ± 9.6 | 0.6 ± 0.2 | 19.1 ± 6.1 | 17.6 ± 10.9 |
|  | P56 | 1.3 ± 0.3* | 18.1 ± 4.2 | 0.2 ± 0.1 | 20.7 ± 4.3 | 6.7 ± 3.3 |

MAR Mineral Apposition Rate

MS Mineralizing Surface

BS Bone Surface

BFR Bone Formation Rate

sL Single Labeled Surface

dL Double Labeled Surface

**Table S3:** The effect of alendronate on trabecular thickness.

| **Group** | **Alendronate Dose** | **Trabecular Thickness (μm)** |
| --- | --- | --- |
| **Normal** | None | Thomopoulos et al, 2007 [5] |
|  | Low (0.125 mg/kg/wk) | 58 ± 7 |
|  | Medium (1.0 mg/kg/wk) | 73 ± 7 |
|  | High (2.0 mg/kg/wk) | 71 ± 7 |
| **Saline** | None | Thomopoulos et al, 2007 [5] |
|  | Low (0.125 mg/kg/wk) | 58 ± 4 |
|  | Medium (1.0 mg/kg/wk) | 51 ± 9 |
|  | High (2.0 mg/kg/wk) | 62 ± 7 |
| **Botox** | None | Thomopoulos et al, 2007 [5] |
|  | Low (0.125 mg/kg/wk) | 57 ± 7 |
|  | Medium (1.0 mg/kg/wk) | 50 ± 9 |
|  | High (2.0 mg/kg/wk) | 61 ± 4 |

**Table S4:** The effect of alendronate on the dynamic histomorphometry of tendon enthesis at P28. Based on an ANOVA, there was a significant effect of dose for MAR, SL/BS, and DL/BS. * p < 0.05 compared to normal at a particular dose.

| **Group** | **Alendronate Dose** | **MAR (μm/day)** | **sL/BS (%)** | **dL/BS (%)** |
| --- | --- | --- | --- | --- |
| **Normal** | None | 4.9 ± 1.7 | 23.3 ± 11.2 | 52.9 ± 16.8 |
|  | Low (0.125 mg/kg/wk) | 4.2 ± 1 | 11.3 ± 5.3 | 72.6 ± 12.9 |
|  | Medium (1.0 mg/kg/wk) | 4.2 ± 0.9 | 24.0 ± 20.1 | 79.4 ± 20.2 |
|  | High (2.0 mg/kg/wk) | 5.6 ± 1.1 | 5.7 ± 1.7 | 90.1 ± 5.2 |
| **Saline** | None | 4.1 ± 0.9 | 33.6 ± 20.4 | 53.5 ± 32.9 |
|  | Low | 3.5 ± 1.1 | 10.9 ± 5.1 | 85.7 ± 8.5 |
|  | Medium | 6.1 ± 1.3* | 25.8 ± 11.8 | 71.8 ± 18.7 |
|  | High | 3.1 ± 1.1* | n.d. | 87.1 ± 6.5 |
| **Botox** | None | 4.3 ± 1.6 | 21.8 ± 16.5 | 46.9 ± 15.9 |
|  | Low | 4.2 ± 1.3 | 15.3 ± 3.8 | 86.3 ± 9.2* |
|  | Medium | 6.0 ± 1.5* | 13.8 ± 7.5 | 85.0 ± 10.4 |
|  | High | 4.0 ± 1.3* | n.d. | 90.1 ± 2.9 |

MAR Mineral Apposition Rate

BS Bone Surface

sL Single Labeled Surface

dL Double Labeled Surface

**Table S5:** The effect of alendronate on the dynamic histomorphometry of trabecular bone directly adjacent to tendon enthesis at P28. Based on an ANOVA, there was a significant effect of dose for MAR, MS/BS, BFR/BS, SL/BS, DL/BS and a significant effect of group for MAR, MS/BS, BFR/BS, SL/BS. * p < 0.05 compared to normal at a particular dose.

| **Group** | **Alendronate Dose** | **MAR (μm/day)** | **MS/BS (%)** | **BFR/BS** | **sL/BS (%)** | **dL/BS (%)** |
| --- | --- | --- | --- | --- | --- | --- |
| **Normal** | None | 2.2 ± 0.4 | 25.4 ± 7.5 | 0.6 ± 0.2 | 17.8 ± 6.1 | 16.5 ± 5.5 |
|  | Low (0.125 mg/kg/wk) | 1.9 ± 0.2 | 29.9 ± 9.4 | 0.6 ± 0.2 | 33.0 ± 11.7 | 13.3 ± 5.8 |
|  | Medium (1.0 mg/kg/wk) | 2.2 ± 0.2 | 37.5 ± 5.9 | 0.8 ± 0.2 | 35.8 ± 5.3 | 19.5 ± 6.2 |
|  | High (2.0 mg/kg/wk) | 2.0 ± 0.2 | 35.2 ± 9.5 | 0.7 ± 0.2 | 43.1 ± 11.9 | 13.7 ± 5.5 |
| **Saline** | None | 2.0 ± 0.3 | 26.2 ± 4.6 | 0.5 ± 0.1 | 16.0 ± 7.2 | 17.6 ± 6.9 |
|  | Low (0.125 mg/kg/wk) | 1.8 ± 0.2 | 36.3 ± 9.8 | 0.7 ± 0.2 | 39.0 ± 10.9 | 16.8 ± 5.1 |
|  | Medium (1.0 mg/kg/wk) | 1.7 ± 0.3* | 22.3 ± 6.5* | 0.4 ± 0.2* | 30.3 ± 8.1 | 7.2 ± 3.4* |
|  | High (2.0 mg/kg/wk) | 1.8 ± 0.3 | 34.4 ± 5 | 0.6 ± 0.1 | 47.5 ± 6.1 | 10.7 ± 3.4 |
| **Botox** | None | 2.0 ± 0.4 | 27.1 ± 9.6 | 0.6 ± 0.2 | 19.1 ± 6.1 | 17.6 ± 10.9 |
|  | Low (0.125 mg/kg/wk) | 1.7 ± 0.3 | 41.4 ± 8.0* | 0.7 ± 0.2 | 49.8 ± 13.9* | 16.5 ± 5.4 |
|  | Medium (1.0 mg/kg/wk) | 2.0 ± 0.2 | 30.5 ± 6.8 | 0.6 ± 0.1* | 39.0 ± 6.4 | 11 ± 4.5* |
|  | High (2.0 mg/kg/wk) | 1.7 ± 0.3* | 42.9 ± 12.2 | 0.7 ± 0.2 | 55.0 ± 14.7* | 15.4 ± 7.2 |

MAR Mineral Apposition Rate

MS Mineralizing Surface

BS Bone Surface

BFR Bone Formation Rate

sL Single Labeled Surface

dL Double Labeled Surface
